# COVID-19 in Coincidence with Transient Distal Renal Tubular Acidosis in an Infant

**DOI:** 10.1155/2022/5361305

**Published:** 2022-05-27

**Authors:** Seyedeh-Kiana Razavi-Amoli, Hamid Mohammadjafari, Daniel Zamanfar, Mohammad Reza Navaeifar, Zahra Sadati-Lamradi, Mohammad Sadegh Rezai

**Affiliations:** ^1^Student Research Committee School of Medicine, Mazandaran University of Medical Sciences, Sari, Iran; ^2^Pediatric Infectious Diseases Research Center, Communicable Diseases Institute, Mazandaran University of Medical Sciences, Sari, Iran; ^3^Diabetes Research Center, Mazandaran University of Medical Sciences, Sari, Iran

## Abstract

**Background:**

Post-COVID-19 nephropathies have been reported profusely in the literature with diverse pathophysiological mechanisms. To the best of our knowledge, this is the first report of transient distal (type 1) renal tubular acidosis (dRTA) in an infant with confirmed COVID-19. *Case Presentation*. We describe a 32-day-old female with diarrhea and fever without respiratory complaints. Her weight, height, and head circumference were normal for age. The primary lab test showed leukocytosis, neutrophilia, elevated inflammatory markers, and non-anion-gap metabolic acidosis. Real-time polymerase chain reaction (RT-PCR) and elevated SARS-CoV-2 immunoglobulin *M* confirmed COVID-19, while echocardiography and spiral chest computed tomography scan were normal. Intravenous fluid therapy and supportive care were initiated. Blood culture was positive for *Klebsiella pneumoniae*. Amikacin and cefotaxime were ordered. Although diarrhea and dehydration gradually improved, venous blood gas still showed metabolic acidosis. Due to the alkaline urine and hypokalemic-hyperchloremic metabolic acidosis, dRTA was diagnosed. Notably, the patient dramatically responded to Shohl's solution.

**Conclusions:**

Regarding the various manifestations of COVID-19, the possible association between dRTA and COVID-19 needs further investigation in children.

## 1. Introduction

Severe acute respiratory syndrome coronavirus 2 (SARS-CoV-2) infection has emerged as a global health concern since it was ﬁrst reported in China in late 2019 [1]. Although clinical presentations of SARS-CoV-2 were presumed to be mild in children [2, 3], the multisystem inﬂammatory syndrome in children (MIS-C) has emerged as one of the life-threatening phenomena with multiorgan involvement, possibly attributed to coronavirus disease 2019 (COVID-19) [4, 5]. COVID-19-associated nephropathies have been reported enormously in the literature with diverse possible pathophysiological mechanisms [6]. Distal (type 1) renal tubular acidosis (dRTA) is defined as the inability to secrete hydrogen ions from the distal tubules and is characterized by hypokalemic- hyperchloremic metabolic acidosis with a normal anion gap and high urine pH [7]. Of importance, transient renal tubular acidosis as a rare manifestation of COVID-19 has been reported in several papers [8, 9]. To the best of our knowledge, this is the first case of COVID-19 in coincidence with transient dRTA in an infant. In this context, we reported a confirmed case of COVID-19 possibly associated with dRTA. This case is being described for its uniqueness since dRTA is seldom attributed to acquired circumstances like infections in children. Of special consideration, it is important to highlight the necessity of pondering COVID-19-related nephropathy in the differential diagnosis of metabolic acidosis in the era of the COVID-19 pandemic.

## 2. Case Presentation

A 32-day-old girl presented with a 4-day history of watery diarrhea and fever. She had 12-13 episodes of watery stools without mucus and blood over the last 24 hours. Notably, there was no history of vomiting, loss of appetite, poor feeding, and respiratory complaints. She had a history of contact with a COVID-19-confirmed case 4 weeks ago. Other family members had no similar presentation. The infant was breastfed since birth, born of the nonconsanguineous marriage, and second in birth order. Her birth weight was 2700 grams (3–10 percentiles). Of importance, no degree of prematurity was noted. Upon admission, she was toxic, tachypneic, and irritable with a temperature of 38.4^o^C (axillary), pulse rate of 140 beats/minute, respiratory rate of 30 beats/minute, and blood pressure of 90/60 mmHg. Her weight, height, and head circumference were normal for her age. Tenting skin turgor, sunken eyes, and soft fontanelle were detected in physical examination. Other physical examinations were unremarkable. Her reflexes and development were normal. She was hospitalized with an initial diagnosis of sepsis and/or COVID-19. Plans for sepsis work-up including analysis of blood, urine, and cerebrospinal fluid were performed. Empirical antibiotic therapy with ampicillin, cefotaxime, and zinc gluconate and intravenous fluid therapy were promptly initiated. Primary laboratory investigation revealed leukocytosis, neutrophilia, and elevated erythrocyte sedimentation rate, C-reactive protein, ammonia, and D-dimer levels. Laboratory results are summarized in Table 1. Venous blood gas (VBG) showed metabolic acidosis (pH: 7.22, HCO_3_−: 11.5 meq/L). Blood sugar and liver function tests were within normal limits. Rehydration therapy and sodium bicarbonate were administrated to correct acidemia. Urine analysis and stool exam were normal. Urine was negative for glucose, acid ammine, ketone, lactate, and reducing substances. At the end of the first admission day, her fever improved but diarrhea was still noted. On the second day, VBG revealed metabolic acidosis (pH: 7.26, HCO_3_−: 11.5 meq/L) with a normal anion gap (11 meq/L). Spiral chest computed tomography (CT) and echocardiography revealed normal levels. To exclude the anatomic abnormality of the urinary system, abdominopelvic ultrasonography was performed which revealed to be normal. Urine and stool cultures were negative, while blood culture was positive for *Klebsiella pneumoniae*. Amikacin was prescribed. Notably, nasopharyngeal real-time polymerase chain reaction (RT-PCR) was positive for SARS-CoV-2, and COVID-19 immunoglobulin *M* (Ig*M*) was elevated (4.5 U). On the fourth day, although diarrhea improved gradually during hospitalization, VBG still showed hyperchloremic metabolic acidosis (pH: 7.21, HCO_3_−: 15 meq/L, chloride: 115 meq/L). Urine pH was 6.5, with a sodium level of 105 mEq/L and specific gravity of 1.012. Furthermore, urine anion gap was positive. Hypokalemia was observed (3 mEq/L). The serum level of ammonia was 235 mg/dhL, for which the normal range is 15–55. Shohl's solution, an alkalinizing citrate used to decrease high levels of acid in blood, was initiated for her. Moreover, sodium benzoate, biotin, and L-carnitine were prescribed to treat elevated ammonia levels. High-performance liquid chromatography (HPLC) was performed to measure serum and urine amino acids levels to consider metabolic disorders which revealed normal results. After resolution of hypovolemic state with aggressive and prompt rehydration therapy, distal renal tubular acidosis was diagnosed according to hypokalemic-hyperchloremic metabolic acidosis with a normal anion gap, positive urine anion gap, and high urine pH. On the 8^th^ day, the patient was discharged with good general condition and recommended to take sodium benzoate, biotin, L-carnitine, and Shohl's solution for a week. The one-month follow-up after discharge showed normal parameters. During follow-up, the patient was not prescribed to continue the described medication.

## 3. Discussion

This report of an exceptional case describes the clinical course of a 32-day-old infant diagnosed with transient dRTA in coincidence with COVID-19 and *Klebsiella* bacteremia. Infants are perceived to have a high incidence of Gram-negative bacteremia, and *Klebsiella* pneumonia is considered to be more common in diarrheal children [10]. Regarding enteric loss of bicarbonates, watery diarrhea often correlates with metabolic acidosis [11]. In our case, metabolic acidosis could be justified due to the illness course and sepsis, but persistent hyperchloremic metabolic acidosis, despite dehydration correction with typical findings including normal anion gap, alkaline urine (pH > 5.5), hypokalemia, and dramatic response to alkali, encouraged us to diagnose dRTA.

Proximal renal tubular acidosis was ruled out of the differential diagnoses due to the fractional HCO_3_− excretion of less than 5%, urine pH > 5.5, and absence of glucose and acid ammine in urine (Fanconi's syndrome).

dRTA or classic RTA is defined as the most common type of RTA and is caused by disrupted renal acidification. In children, dRTA is almost perceived as a primary hereditary disease in both autosomal recessive and autosomal dominant patterns with genetic causes comprising at least three genes: ATP6V1B1, ATP6V0A4, and SLC4A1 [12, 13]. Autosomal recessive dRTA mostly presents in the first months of life with manifestations including nephrocalcinosis and hearing loss. Autosomal dominant dRTA is considered to be less severe and occurs during adolescence or adulthood [7]. In the current patient, we could not identify other sources of secondary dRTA including medications, toxins, and urological abnormalities that propose the likelihood of primary dRTA. Nevertheless, evaluations of mutations and autoimmune disorders were not performed to ensure this assumption.

Consistent with several reports [8, 9], we hypothesized that COVID-19 could potentially cause or trigger dRTA in this patient. As the exact mechanism of COVID-19-related nephropathy is still not clear, the association of dRTA and COVID-19 in the current patient could seem coincidental.

COVID-19-associated nephropathies may occur due to diverse mechanisms including ischemic injury, cytokine storm, and direct SARS-CoV-2 injury. Moreover, the presence of angiotensin-converting enzyme 2 (ACE 2) as a receptor for SARS-CoV-2 cellular entry in the brush borders of renal tubular epithelial cells potentially makes sure of direct invasion of SARS-CoV-2 in the kidneys [14].

Furthermore, in the era of COVID-19, MIS-C must be considered in children with fever lasting more than 72 hours, and at least involving two organ systems, elevated markers of inflammation without other microbial causes, and evidence of COVID-19 [4]. In our case, obvious bacterial sepsis reasonably led us to exclude MIS-C.

Hyperammonemia in our patient necessitated the evaluation of metabolic disorder, an inborn error of metabolism, and liver dysfunction. Metabolic disorders were ruled out due to the absence of ketones, glucose, and organic acid in urine. Moreover, blood concentrations of glucose, lactate, pyruvate, and amino acid were in the normal range. Therefore, an inborn error of metabolism could be considerately excluded. Furthermore, normal liver enzymes, albumin, serum bilirubin, and partial thrombin time reasonably excluded liver dysfunction. Consistent with a study by Palmer et al. [15], hyperammonemia may occur during metabolic decompensation as a result of an imbalance between the increased ammonia synthesis, in response to metabolic acidosis, and the impaired ammonia excretion in some cases of dRTA.

Serum calcium level was in the normal range in our infant. However, a study by Fuster et al. [16] has shown that patients with dRTA are in negative calcium balance due to bone resorption to buffer chronic metabolic acidosis. Therefore, it may cause rickets and nephrocalcinosis in untreated dRTA.

Management of dRTA includes supplementation therapy with sodium bicarbonate and potassium. Early diagnosis of dRTA is pivotal to prevent severe complications due to hypokalemia including progressive muscle weakness and paralysis.

## 4. Conclusion

The current exceptional case report emphasizes that with regards to the substantial heterogeneity in COVID-19 manifestations, we have to take into consideration infection-related nephropathy in the differential diagnosis of metabolic acidosis in the era of the COVID-19 surge. Further evaluation regarding the association between dRTA and infections in children is warranted.

## Figures and Tables

**Table 1:** Laboratory tests of the patient during hospitalization.

1^st^ day	2^nd^ day	4^th^ day
WBC: 20.14 (×10^3^ u/L)	WBC: 15.6 (×10^3^ u/L)	WBC: 10.1 (×10^3^ u/L)
Neut: 57%	Neut: 49%	Potassium: 3 (meq/l)
Lymph: 22%	Lymph: 39%	Cl: 115 (meq/l)
Hemoglobin: 14.8 (gr/dl)	PLT: 393 (×10^3^ u/L)	Na: 138 (meq/l)
PLT: 467 (×10^3^ u/L)	Urea: 12	Ca: 10.2 (meq/l)
Blood sugar: 81 (mg/dl)	Creatinine: 0.4 (mg/dl)	Urea: 6.5
Troponin: 0.1 (IU/ml)	Na: 144 (meq/l)	Creatinine: 0.3 (mg/dl)
ESR: 118 (mm/hr); CRP: 51 (mg/L)	Potassium: 3.2 (meq/l)	Urine analysis: pH = 6; SG = 1.012; Na: 105
Ammonia: 230 (g/dl) (normal range: 15–55)	Ammonia: 209 (g/dl)	Ammonia: 235 (g/dl)
Protein total: 7.3 (g/h)	CRP: 9 (mg/L)	ESR: 22 (mm/hr)
Albumin: 4.8	AST: 52 (U/l)	CRP: 7 (mg/L)
Creatinine: 0.6 (mg/dl)	ALT: 8 (U/l)	Prothrombin time: 12 (hr)
Lactate: 16 (mg/dl)	ESR: 38 (mm/hr)	Partial thromboplastin time: 25 (hr)
Potassium: 3.5 (meq/l)	Protein total: 5 (g/h)	Bilirubin: 1 (mg/dl)
Na: 137 (meq/l)	Albumin: 3.4	Blood sugar: 80 (mg/dl)

## Data Availability

Datasets used during the current study are available from the corresponding author on reasonable request (drmsrezaii@yahoo.com).
